# Relationship between serum uric acid and estimated glomerular filtration rate in adolescents aged 12-19 years with different body mass indices: a cross-sectional study

**DOI:** 10.3389/fendo.2023.1138513

**Published:** 2023-07-26

**Authors:** Qiuwei Tian, Caixia He, Zisai Wang, Marady Hun, Yi-Cheng Fu, Mingyi Zhao, Qingnan He

**Affiliations:** ^1^ Department of Pediatrics, The Third Xiangya Hospital, Central South University, Changsha, China; ^2^ Department of Pediatrics, Renmin Hospital of Wuhan University, Wuhan University, Wuhan, China

**Keywords:** serum uric acid, estimated glomerular filtration rate, BMI, adolescents, NHANES

## Abstract

**Background:**

Globally, chronic kidney disease (CKD) is a growing public health concern. Serum uric acid (SUA) is an easily detectable and readily available biochemical indicator that has long been recognized as an independent risk factor for CKD. In addition, studies have indicated a potential relationship between SUA and body mass index (BMI). However, studies on the effect of SUA levels on the estimated glomerular filtration rate (eGFR) in adolescents with different BMIs are very rare.

**Methods:**

Weighted multiple regression analysis was used to estimate the independent relationship between SUA and log-transformed eGFR. Additionally, we used a weighted generalized additive model and smooth curve fitting to describe the nonlinear relationships in the subgroup analysis.

**Results:**

First, SUA was negatively associated with log-transformed eGFR even after adjusting for all covariates (β=-0.0177, 95% CI: -0.0203-0.0151, P<0.0001). Second, the results of the stratified analysis found that after adjusting for all covariates, the decrease in log-transformed eGFR due to changes in per SUA levels (Per 1, mg/dL increase) was elevated in female adolescents (β=-0.0177, 95% CI: -0.0216, -0.0138, P<0.0001), adolescents aged 12-15 years (β=-0.0163, 95% CI: -0.0200, -0.0125, P<0.0001) and black (β=-0.0199, 95% CI: -0.0251, -0.0148, P<0.0001) adolescents. Furthermore, we found that adolescents with a higher BMI had higher SUA levels, and the effect of SUA on eGFR was significantly higher in underweight adolescents (β=-0.0386, 95% CI: (-0.0550, -0.0223), P<0.0001).

**Conclusion:**

SUA was negatively associated with the eGFR in adolescents aged 12-19 years. Furthermore, we found for the first time that SUA affects the eGFR differently in adolescents with different BMIs. This effect was particularly significant in underweight adolescents.

## Introduction

Chronic kidney disease (CKD) is a growing global public health problem. Over the nearly 30 years from 1990 to 2017, the global prevalence of CKD at all ages increased by 29.3%, while the age-standardized prevalence remained stable (1). Previous studies on adults have found that both poor lifestyle habits, such as smoking, alcohol consumption and sedentary lifestyle, as well as a polluted atmosphere, such as elevated fine particulate matter (PM2.5) in the air, and even low birth weight in infants can be risk factors for the development and progression of CKD (2–4). Although epidemiological studies on CKD in adolescents are very limited, it is indisputable that CKD in children and adolescents has become one of the most significant diseases affecting their lives. Therefore, it is critical to find a biomarker that facilitates early prediction and timely intervention by clinicians for the prevention of CKD in adolescents.

It is well known that uric acid is the final product of purine metabolism. As a clinically easily detectable and available biochemical indicator, SUA is closely associated with diseases such as hypertension (5–7), diabetes (7–10), and metabolic syndrome (11, 12) and has been recognized as an independent risk factor for the development of CKD (13, 14). Meanwhile, SUA levels are also correlated with body mass index (BMI). For example, some scholars have found a nonlinear relationship between BMI and SUA in adults (15). In addition, studies in obese patients have also found that elevated SUA levels are always accompanied by obesity in both adults and adolescents (16, 17). This may be related to oxidative stress and the inflammatory response induced by xanthine oxidoreductase-derived reactive oxygen species and uric acid (18, 19). Therefore, based on the findings of previous studies, we hypothesized that controlling BMI might enable the modification of SUA levels in adolescents.

Previous findings have indicated that serum uric acid can cause renal injury through both crystal-dependent and crystal-independent mechanisms (20, 21). Therefore, in this study, we used the estimated glomerular filtration rate (eGFR) as a measure of basal renal function in adolescents based on a cross-sectional survey from the National Health and Nutrition Examination Survey (NHANES) database with the aim of exploring two main questions. First, we investigated the relationship between SUA and eGFR in adolescents in a cross-sectional survey with a large sample size. Second, we investigated the effect of SUA levels on renal function in adolescents with different BMIs, with the aim of exploring whether the renal injury effect of SUA can be reduced by controlling the BMI of adolescents, thus preventing the occurrence of CKD in adolescents.

## Materials and methods

### Study population

The National Health and Nutrition Examination Survey (NHANES) is a large cross-sectional survey based on the U.S. population with a two-year survey cycle. This survey collected a large amount of survey information from the general U.S. population through a complex, multistage, probability sampling design. All adult participants provided written informed consent, and participants under 18 years of age were required to submit the consent of their parents or guardians. For data researchers and users, the survey data from NHANES are publicly available at www.cdc.gov/nchs/nhanes/. NHANES has been reviewed by the National Center for Health Statistics Research Ethics Review Board.

**Figure 1:**
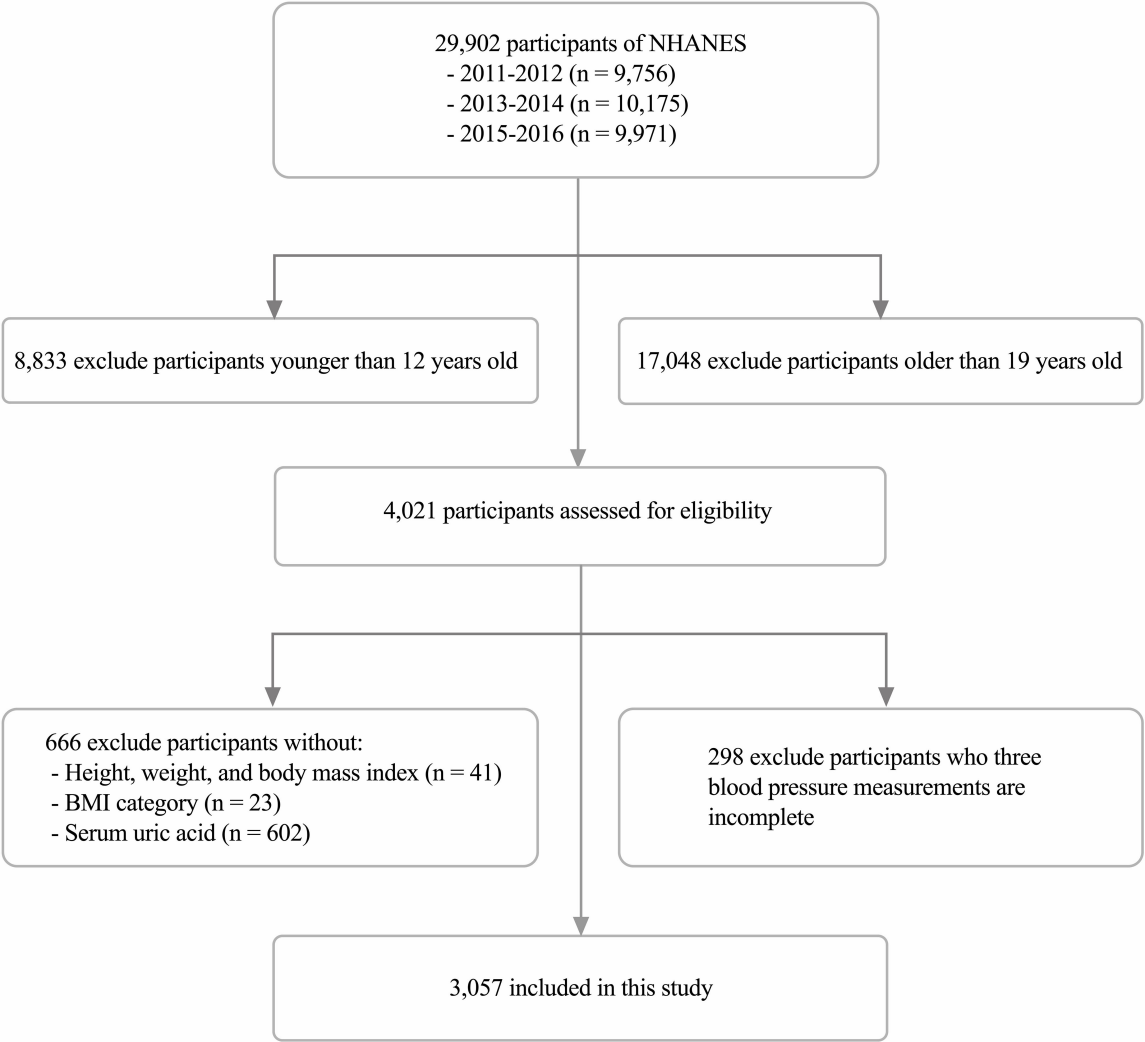
Flow of participants through the study.

Our study collected relevant data, including demographics, physical examinations, laboratory tests, and questionnaires, during the three survey cycles of NHANES 2011-2016 and analyzed them. The inclusion and exclusion details of the study population in this study are shown in **Figure 1**.

### Variables

The exposure variable in this study was SUA. The DxC800 uses a timed endpoint method to measure the concentration of uric acid in serum, plasma or urine. The outcome variable was the estimated glomerular filtration rate (eGFR), calculated using the latest modified Schwartz equation: eGFR=0.413*[height (cm)/serum creatinine (mg/dL)] (22). The body measurement data were collected in the Mobile Examination Center (MEC) by trained health technicians. The poverty income ratio is the ratio of family income to poverty and is calculated using the Department of Health and Human Services (HHS) poverty guidelines as the poverty measure. The scale includes the following terms and corresponding values: “mild” (poverty income ratio <1.99), “moderate” (1.99 ≤ poverty income ratio ≤ 3.49), and “severe” (poverty income ratio > 3.49). The BMI (body mass index), expressed as weight in kilograms divided by height in meters squared (kg/m2), is commonly used to classify weight status. The definitions of underweight, normal weight, overweight, and obesity in children and adolescents are not directly comparable with the definitions in adults, which were defined as “underweight” (BMI < 5th percentile), “normal weight” (BMI 5th to < 85th percentiles), “overweight” (BMI 85th to < 95th percentiles) and “obese” (BMI ≥ 95th percentile) (23). Some details of other incorporated variables are openly available at www.cdc.gov/nchs/nhanes/.

Based on previous relevant studies, clinical experience, and data collected in the NHANES database, the continuous variables among the covariates included in this study were age, weight, waist circumference, BMI, systolic blood pressure (SBP), diastolic blood pressure (DBP), albumin, globulin, total protein, alanine aminotransferase (ALT), aspartate aminotransferase (AST), gamma glutamyl transaminase (GGT), lactate dehydrogenase (LDH), blood urea nitrogen (BUN), serum glucose, total cholesterol, triglycerides, low-density lipoprotein (LDL), high-density lipoprotein (HDL), and urinary albumin creatinine ratio (ACR). The categorical variables among the covariates were sex, race, education level, poverty income ratio, and BMI category. In addition, height and serum creatinine were not included as covariates because of their involvement in the calculation of the eGFR.

### Data analysis

Data analysis for this study was completed under the guidance of the CDC guidelines. Furthermore, given the complexity and nonresponse of the NHANES survey design, we used sample weights to analyze the data. Because of the skewed distribution of the eGFR, we used the log-transformed eGFR (LgeGFR) for the analysis of the eGFR. First, the sample weights were used in the analysis of weighted means (continuous variables), percentages (categorical variables), and standard errors of baseline characteristics. Second, after adjustment for potential confounders, weighted multiple regression analysis was used to estimate the independent relationship between SUA and LgeGFR. Third, weighted generalized additive models and smooth curve fitting were used to describe the nonlinear relationships in the subgroup analysis. Three models were used in the regression analysis: Model 1 was the crude model and did not include any covariates for adjustment; Model 2 was adjusted for age, sex, race, and BMI; Model 3 was adjusted for age, sex, race, education attainment, poverty income ratio, weight, waist circumference, BMI, BMI category, SBP, DBP, albumin, globulin, total protein, ALT, AST, GGT, LDH, BUN, serum glucose, total cholesterol, triglycerides, LDL, HDL, and the ACR.

Categorical variables are expressed as frequencies or percentages, and continuous variables are expressed as the means ± standard deviations. All data analyses were performed using the R package data software (http://www.R-project.org) and Empower (http://www.empowerstats.com). P < 0.05 was considered statistically significant.

## Results

### Description of baseline characteristics

The weighted demographic and medical characteristics are described in **Table 1**. A total of 3057 adolescents aged 12-19 years were included in our study, of whom 51.88% were male, 48.12% were female, 55.17% were white, 13.84% were black, 14.40% were Mexican American, and 16.60% were of other races. Significant differences were found in each of the baseline characteristics of the groups according to the four subgroups of SUA, except for poverty income ratio, globulin and total cholesterol. In addition, participants with SUA in the lower range (Q1: <4.00 mg/dL, Q2: 4.10-4.80 mg/dL) were more likely to be female (79.67%, 69.51%), while those in the higher range (Q3: 4.90-5.70 mg/dL, Q4: ≥5.80 mg/dL) were more likely to be male (64.32%, 81.21%). Compared to other subgroups, participants in the top quartile of SUA levels had a higher weight, height, waist circumference, BMI, SBP, DBP, albumin, ALT, AST, LDH, BUN, serum creatinine, serum glucose, total cholesterol, triglycerides, and LDL and lower levels of HDL and eGFR. Additionally, we observed that participants in the lowest quartile of SUA levels had much higher urinary albumin creatinine ratios than those in the other groups.

**Table 1:** Description of 3,057 participants included in the present study.

Characteristics	Serum uric acid, mg/dL					
	Total	Q1 (<4.00)	Q2 (4.10-4.80)	Q3 (4.90-5.70)	Q4 (≥5.80)	P value
n	3057	735	759	769	794	
Age, years	15.35 ± 2.23	15.08 ± 2.22	15.09 ± 2.22	15.28 ± 2.22	15.89 ± 2.15	<0.0001
Sex (%)						<0.0001
Male	51.88	20.33	34.49	64.32	81.21	
Female	48.12	79.67	65.51	35.68	18.79	
Race (%)						0.0015
White	55.17	48.11	55.68	58	57.64	
Black	13.84	18.39	14.22	12.25	11.35	
Mexican American	14.4	16.22	14.5	13.05	14.15	
Other	16.6	17.28	15.6	16.69	16.86	
Education attainment (%)						0.001
Less than high school	85.31	87.95	87.97	85.24	80.81	
High school	8.51	7.69	7.86	7.96	10.3	
Higher than high school	6.15	4.36	4.17	6.8	8.79	
Other	0.03				0.1	
Poverty income ratio (%)						0.4619
Low	29.51	31.16	29.24	28.6	29.29	
Middle	36.67	34.91	38.07	39.13	34.41	
High	27.9	27.78	26.11	26.98	30.55	
Not recorded	5.92	6.15	6.58	5.28	5.75	
Weight (Kg)	66.47 ± 19.83	56.92 ± 14.73	60.65 ± 14.66	66.03 ± 16.62	80.00 ± 22.94	<0.0001
Height (cm)	165.62 ± 9.88	160.46 ± 8.26	162.66 ± 8.47	166.91 ± 9.67	171.26 ± 9.28	<0.0001
Waist circumference (cm)	82.25 ± 14.92	76.64 ± 11.33	79.00 ± 12.20	81.59 ± 13.64	90.43 ± 17.33	<0.0001
BMI (kg/m2)	24.05 ± 6.19	21.98 ± 5.01	22.86 ± 5.04	23.66 ± 5.54	27.21 ± 7.30	<0.0001
BMI category (%)						<0.0001
Underweight	3.71	4.65	4.41	3.63	2.38	
Healthy weight	58.5	73	63.73	60.04	40.4	
Overweight	17.61	13.75	17.8	18.49	19.71	
Obese	20.18	8.61	14.05	17.84	37.51	
SBP (mmHg)	109.17 ± 9.53	106.39 ± 8.59	107.14 ± 8.78	109.70 ± 9.67	112.77 ± 9.53	<0.0001
DBP (mmHg)	59.17 ± 11.93	58.69 ± 11.13	59.05 ± 11.75	58.68 ± 12.50	60.16 ± 12.10	0.042
Albumin (g/dL)	4.50 ± 0.30	4.44 ± 0.29	4.46 ± 0.32	4.54 ± 0.30	4.56 ± 0.30	<0.0001
Globulin (g/dL)	2.72 ± 0.38	2.75 ± 0.37	2.70 ± 0.41	2.71 ± 0.37	2.72 ± 0.37	0.0953
Total protein (g/dL)	7.22 ± 0.41	7.19 ± 0.40	7.16 ± 0.40	7.25 ± 0.42	7.28 ± 0.41	<0.0001
ALT (U/L)	19.32 ± 12.34	15.49 ± 5.41	17.78 ± 13.04	18.93 ± 9.99	24.22 ± 15.79	<0.0001
AST (U/L)	23.80 ± 11.14	21.92 ± 5.53	22.55 ± 6.26	24.09 ± 12.22	26.20 ± 15.58	<0.0001
GGT (U/L)	14.33 ± 9.62	11.81 ± 4.27	13.31 ± 12.29	13.84 ± 5.61	17.78 ± 11.89	<0.0001
LDH (U/L)	129.98 ± 29.06	128.12 ± 25.72	128.37 ± 26.34	129.54 ± 27.86	133.40 ± 34.38	0.0008
BUN (mg/dL)	11.07 ± 3.43	10.51 ± 3.15	10.68 ± 3.21	11.21 ± 3.41	11.74 ± 3.73	<0.0001
Creatinine (mg/dL)	0.72 ± 0.17	0.64 ± 0.12	0.68 ± 0.14	0.75 ± 0.17	0.81 ± 0.18	<0.0001
Serum glucose (mg/dL)	88.85 ± 14.57	89.05 ± 13.87	88.81 ± 19.56	87.60 ± 9.88	89.94 ± 13.51	0.0134
Cholesterol (mg/dL)	157.51 ± 29.52	158.25 ± 27.12	156.81 ± 29.10	156.10 ± 29.23	158.93 ± 31.89	0.2027
Triglycerides (mg/dL)	99.27 ± 71.88	83.51 ± 54.80	93.45 ± 57.45	99.10 ± 70.24	117.58 ± 91.07	<0.0001
LDL (mg/dL)	86.93 ± 17.50	85.07 ± 15.24	86.63 ± 16.85	87.44 ± 17.97	88.21 ± 19.12	0.0049
HDL (mg/dL)	51.56 ± 12.12	56.62 ± 13.08	52.99 ± 11.19	51.10 ± 11.35	46.57 ± 10.80	<0.0001
ACR (mg/g)	26.97 ± 110.82	41.16 ± 153.41	26.42 ± 83.06	20.20 ± 94.88	22.61 ± 105.08	0.0016
eGFR (ml/min/1.73m^2^)	98.73 ± 20.27	107.60 ± 19.63	102.24 ± 18.83	96.11 ± 20.32	90.89 ± 18.40	<0.0001
LgeGFR (ml/min/1.73m^2^)	1.99 ± 0.09	2.02 ± 0.08	2.00 ± 0.08	1.97 ± 0.09	1.95 ± 0.09	<0.0001

BMI, body mass index; SBP, systolic blood pressure; DBP, diastolic blood pressure; ALT, alanine aminotransferase; AST, Aspartate Aminotransferase; GGT, Gamma glutamyl Transaminase; LDH, Lactate Dehydrogenase; BUN, Blood Urea Nitrogen; LDL, low-density lipoprotein; HDL, high-density lipoprotein; SUA, serum uric acid; ACR, albumin creatinine ratio; eGFR, estimated glomerular filtration rate. Mean ± s.d. for continuous variables: P value was calculated using a weighted linear regression model. % for Categorical variables: P value was calculated by weighted chi-square test.

### Relationship between SUA and log-transformed eGFR

**Figure 2:**
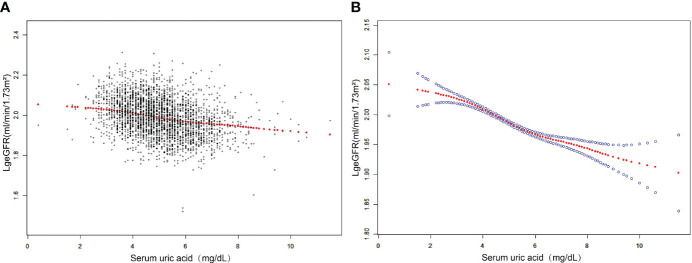
The association between SUA and log-transformed eGFR. **(A)** Each black point represents a sample. **(B)** The solid red line represents the smooth curve fit between variables. Blue bands represent the 95% confidence interval from the fit. Adjusted for age, sex, race, education attainment, poverty income ratio, weight, waist circumference, BMI, BMI category, SBP, DBP, albumin, globulin, total protein, ALT, AST, GGT, LDH, blood urea nitrogen, serum glucose, cholesterol, triglycerides, LDL, HDL, albumin creatinine ratio.

The results of the multivariate regression analysis are shown in **Table 2**, and the smoothed curve fits and scatter plots are shown in **Figure 2**. When not adjusted for any covariates (Model 1), SUA was negatively associated with log-transformed eGFR (β=-0.0232, 95% CI: -0.0256-0.0207, P<0.0001). After adjusting for age, sex, race, and BMI category only (Model 2) (β=-0.0177, 95% CI: -0.0204- -0.0151, P<0.0001) and after adjusting for all covariates (Model 3) (β=-0.0177, 95% CI: -0.0203-0.0151, P<0.0001), this negative correlation remained. In addition, we found that compared to baseline levels in the lowest quartile of SUA, the log-transformed eGFR of the group in the highest quartile was 0.0508 lg (ml/min/1.73 m2) lower than that of the lowest quartile. Meanwhile, P for trend test all had P<0.001, suggesting that the trend of decreased LgeGFR was significant for each increase in SUA level.

**Table 2:** Association of SUA with LgeGFR among 3,057 12–19 year-old adolescents, NHANES 2011–2016.

	Model1β (95%CI) P value	Model2 β (95%CI) P value	Model3β (95%CI) P value
**Serum uric acid**	-0.0232 (-0.0256, -0.0207) <0.0001	-0.0177 (-0.0204, -0.0151) <0.0001	-0.0177 (-0.0203, -0.0151) <0.0001
SUA categories			
Q1(0.40-4.00 mg/dL)	Reference	Reference	Reference
Q2(4.10-4.80 mg/dL)	-0.0223(-0.0310, -0.0136) <0.0001	-0.0205 (-0.0281, -0.0129) <0.0001	-0.0173 (-0.0244, -0.0101) <0.0001
Q3(4.90-5.70 mg/dL)	-0.0514(-0.0600, -0.0428) <0.0001	-0.0403 (-0.0483, -0.0323) <0.0001	-0.0372 (-0.0448, -0.0296) <0.0001
Q4(5.80-11.50 mg/dL)	-0.0748(-0.0833, -0.0662) <0.0001	-0.0544 (-0.0633, -0.0456) <0.0001	-0.0508 (-0.0593, -0.0422) <0.0001
P for trend	<0.001	<0.001	<0.001

Model 1, no covariates were adjusted.

Model 2, adjust for age, sex, race and BMI category.

Model 3, adjust for age, sex, race, education attainment, poverty income ratio, weight (Smooth), waist circumference, BMI, BMI category, SBP, DBP, albumin, globulin, total protein, ALT, AST, GGT, LDH, blood urea nitrogen, serum glucose, cholesterol, triglycerides, LDL, HDL, albumin creatinine ratio. Generalized additive models were applied.

### Subgroup analysis based on potential effects

**Figure 3:**
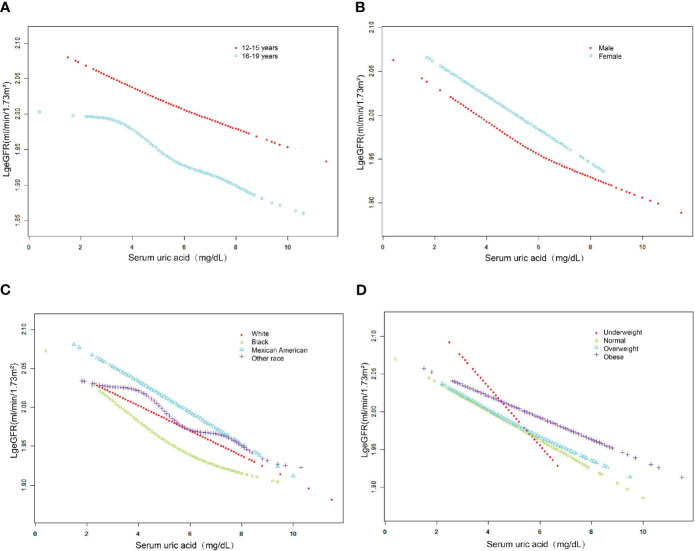
The association between serum uric acid and log-transformed eGFR stratified by age **(A)**, sex **(B)**, race **(C)** and BMI category **(D)**. Adjusted for education attainment, poverty income ratio, weight, waist circumference, BMI, SBP, DBP, albumin, globulin, total protein, ALT, AST, GGT, LDH, blood urea nitrogen, serum glucose, cholesterol, triglycerides, LDL, HDL, albumin creatinine ratio.

Subgroup analysis according to sex, age, race, and BMI category is shown in **Table 3**. After subgroup analysis by sex, age, and race, male adolescents (SUA 5.60 ± 1.15 mg/dL), adolescents aged 16-19 years (SUA 5.19 ± 1.27 mg/dL), and white adolescents (SUA 5.12 ± 1.20 mg/dL) had higher SUA levels. After adjustment for all covariates (Model 3), the decrease in log-transformed eGFR due to changes in SUA levels (Per 1, mg/dL increase) was higher in female adolescents (β=-0.0177, 95% CI: -0.0216, -0.0138, P<0.0001), adolescents aged 12-15 years (β=- 0.0163, 95% CI: -0.0200, -0.0125, P<0.0001) and black (β=-0.0199, 95% CI: -0.0251, -0.0148, P<0.0001) adolescents. Furthermore, we found that adolescents with higher BMI had higher SUA levels, and the effect of SUA on eGFR was highest in underweight adolescents compared to other body types (β=-0.0386, 95% CI: (-0.0550, -0.0223), P<0.0001). As shown in **Figure 3**, after stratifying by age, sex, race and BMI category, we also attempted to use smoothed curve fitting to find a linear and nonlinear relationship between SUA and log-transformed eGFR in different subgroups. Finally, interaction tests showed that the relationship between SUA and log-transformed eGFR was significantly different (P for interaction < 0.05) between adolescents of different races (P for interaction = 0.015) and different BMI categories (P for interaction = 0.003), while this negative relationship was concordant across subgroups of sex and age.

**Table 3:** Subgroup analyses of the effect of SUA on LgeGFR.

Subgroups	n	Mean ± SD	β (95%CI) P value	P for interaction
stratified by Sex				
Male	1567	5.60 ± 1.15	-0.0148 (-0.0183, -0.0113) <0.0001	0.587
Female	1470	4.46 ± 1.00	-0.0177 (-0.0216, -0.0138) <0.0001	
stratified by Age				
12-15 years	1591	4.93 ± 1.16	-0.0163 (-0.0200, -0.0125) <0.0001	0.961
16-19 years	1466	5.19 ± 1.27	-0.0149 (-0.0185, -0.0114) <0.0001	
stratified by Race				
White	792	5.12 ± 1.20	-0.0171 (-0.0219, -0.0123) <0.0001	0.015
Black	773	4.82 ± 1.23	-0.0199 (-0.0251, -0.0148) <0.0001	
Mexican American	642	4.99 ± 1.24	-0.0154 (-0.0210, -0.0097) <0.0001	
Other race	850	5.06 ± 1.23	-0.0140 (-0.0194, -0.0087) <0.0001	
stratified by BMI category				
Underweight	105	4.70 ± 0.98	-0.0386 (-0.0550, -0.0223) <0.0001	0.003
Normal weight	1760	4.79 ± 1.09	-0.0177 (-0.0213, -0.0141) <0.0001	
Overweight	553	5.17 ± 1.14	-0.0190 (-0.0248, -0.0132) <0.0001	
Obese	639	5.77 ± 1.36	-0.0146 (-0.0201, -0.0091) <0.0001	

Adjust for age, sex, race, education attainment, poverty income ratio, weight, waist circumference, BMI, BMI category, SBP, DBP, albumin, globulin, total protein, ALT, AST, GGT, LDH, blood urea nitrogen, serum glucose, cholesterol, triglycerides, LDL, HDL, albumin creatinine ratio. Mean  ± SD for SUA.

### Log-transformed eGFR by quartiles of SUA, stratified by age and BMI category

As shown in **Table 4**, we stratified adolescents aged 12-19 years by age. The results showed a significant association between SUA levels and lower log-transformed eGFR (P for trend < 0.001) among all normal weight adolescents, underweight adolescents aged 16-19 years, overweight adolescents aged 12-15 years, and obese adolescents among the 3057 participants included after adjustment for age, sex, race, and BMI category. Additionally, the strongest associations were observed in all normal weight adolescents combined, 16- to 19-year-old underweight adolescents, and 12- to 15-year-old overweight adolescents, all of whom had reduced log-transformed eGFR for each SUA quartile. Finally, the results of the trend test were significant (P for trend<0.001) for all subgroups except for underweight adolescents aged 12-15 years and overweight and obese adolescents aged 16-19 years.

**Table 4:** log-transformed eGFR by quartiles of serum uric acid, stratified by age and BMI category.

Quartiles of serum uric acid	LgeGFR (ml/min/1.73m^2^β (95%CI)			
	Underweight	Normal weight	Overweight	Obese
12–15 years				
Q1 (0.40-4.00)	2.0632 (2.0126, 2.1138)	2.0421 (2.0333, 2.0508)	2.0596 (2.0351, 2.0841)	2.0723 (2.0433, 2.1012)
Q2 (4.10-4.80)	2.0725 (2.0243, 2.1207)	2.0253 (2.0168, 2.0337)	2.0347 (2.0151, 2.0543)	2.0291 (2.0085, 2.0497)
Q3 (4.90-5.70)	2.0299 (1.9675, 2.0922)	2.0112 (2.0022, 2.0203)	2.0136 (1.9947, 2.0325)	2.0303 (2.0138, 2.0469)
Q4 (5.80-11.50)	2.0156 (1.9364, 2.0948)	2.0040 (1.9903, 2.0176)	1.9641 (1.9438, 1.9845)	2.0077 (1.9931, 2.0223)
P for trend	0.192	<0.001	<0.001	<0.001
16–19 years				
Q1 (0.40-4.00)	2.0054 (1.9630, 2.0477)	1.9720 (1.9620, 1.9821)	1.9582 (1.9392, 1.9772)	1.9856 (1.9566, 2.0145)
Q2 (4.10-4.80)	2.0110 (1.9736, 2.0485)	1.9495 (1.9397, 1.9593)	1.9607 (1.9437, 1.9777)	1.9604 (1.9359, 1.9849)
Q3 (4.90-5.70)	1.9327 (1.8940, 1.9714)	1.9329 (1.9232, 1.9425)	1.9365 (1.9212, 1.9518)	1.9686 (1.9485, 1.9887)
Q4 (5.80-11.50)	1.9148 (1.8740, 1.9556)	1.9260 (1.9156, 1.9364)	1.9468 (1.9315, 1.9621)	1.9526 (1.9392, 1.9660)
P for trend	<0.001	<0.001	0.233	0.069

Adjust for age, sex, race and BMI category.

## Discussion

In this large population-based and representative cross-sectional study, we found an inverse relationship between SUA and log-transformed eGFR, and the relationship was significantly different among adolescents of different ethnicities and BMI. Notably, compared with adolescents with other BMI categories, we found for the first time that the effect of SUA on log-transformed eGFR was greatest in underweight adolescents. Finally, this study also investigated the conclusions of a previous study that higher SUA levels are associated with higher BMI and that there are sex differences in SUA levels.

Recently, many intensive studies on SUA have found that SUA is an associated risk factor for many diseases (10, 13, 24–26). Previous studies on the effect of SUA on kidney injury have mainly focused on the prognosis of CKD. However, this field is still fraught with controversy. Sampson A.L. et al. performed a systematic review including 12 studies with 1,187 participants. The results indicated that the protective effect of uric acid-lowering therapy on renal function has obvious time-dependent effects. A decrease in serum creatinine and an increase in eGFR were observed after one year of uric acid-lowering therapy, but it showed little or no effect on eGFR after two years (27). However, another study showed different results about an inverse relationship between SUA levels and protection from CKD incidence and progression. Lower UA levels were protective for the risk of CKD incidence (RR 0.65 [95% CI 0.56-0.75]) and progression (RR 0.55 [95% CI 0.44-0.68]) (28). The results that we obtained also support this conclusion. That is, an apparent aggravated kidney injury characterized by a decrease in the eGFR is accompanied by elevated levels of serum uric acid. The reason for this result may be related to crystal-dependent or crystal-independent mechanisms of renal injury associated with SUA (20, 21). Nevertheless, further longitudinal studies are needed to verify these contradictory findings, which can be attributed to the differences in study design, study populations, and adjustment of confounding factors.

The study of uric acid has yielded a wealth of research results over the past few decades, both in the basic and clinical fields. The most discussed mechanisms of uric acid-induced kidney injury include oxidative stress, endothelial cell dysfunction, renal fibrosis, and renal inflammatory response (29). Past studies on the physiological functions of uric acid have found that it can counteract oxidative stress by avoiding oxidative inactivation of endothelial enzymes and by maintaining vascular endothelium-mediated vasodilatation (30). However, uric acid can also exacerbate cell and tissue damage due to oxidative stress by increasing the production of reactive oxygen species. For example, as precursors of uric acid, purines can increase reactive oxygen levels by inducing IFN-γ upregulation of xanthine oxidoreductase expression (31). Excessive production of reactive oxygen species causes cellular damage, including vascular endothelial cells. Meanwhile, high-dose uric acid-mediated reduction in nitric oxide synthase activity and nitric oxide production is also involved in endothelial cell dysfunction, which ultimately increases the incidence of numerous adverse outcome events represented by cardiovascular events (32). More evidence from basic research is needed to confirm the details of this mechanism.

In the current study, we used a combination of subgroup analysis and an interaction test to find different effects of SUA on eGFR in adolescents with different BMIs. In addition, we focused for the first time on underweight adolescents as a potential at-risk population. As mentioned earlier, the effect of SUA levels on kidney function in the population can be influenced by many metabolic diseases and even by poor lifestyle habits such as smoking and alcohol consumption (33–35). Thus, the selection of adolescents can avoid the interference of these potential confounders and make them the ideal population to study.

Baseline data on renal function in underweight adolescents are limited. A study of malnutrition-inflammation-cachexia syndrome (MICS) in older individuals concluded that MICS could influence the relationship between high eGFR and mortality, particularly in people with low body mass index (36). This suggests, to some extent, that underweight adolescents may have an impairment of the glomerular filtration rate itself. Furthermore, although low SUA levels in the underweight adolescents in this study cannot be defined as hypouricemia compared to other adolescents, previous studies have found that renal hypouricemia can lead to the development of kidney stones as well as exercise-induced acute kidney injury (37, 38).

In addition, low weight is often associated with a reduced incidence of hyperuricaemia (39). A recent cohort study based on a large sample size found that SUA<5.7 mg/dL was a key inflection point for predicting mortality risk in the population. The study showed for the first time that muscle loss and weight loss were more common in people with low SUA and were associated with higher mortality (40). A point worth noting for us is that the eGFR, which measured glomerular filtration levels in adolescents in this study, is an imperfect estimate of the true glomerular filtration rate based on creatinine measurements. Among the factors affecting creatinine concentrations are, on the one hand, residual functional clearance from the glomerulus and, on the other hand, indirect conversion of creatine from muscle metabolism. Therefore, muscle mass is a key factor influencing the magnitude of eGFR based on creatinine measurements (41). In the present study, the greatest effect of SUA on glomerular filtration rate was observed in adolescents with a lean BMI, probably due to the influence of low SUA levels in the lean adolescents themselves, and on the other hand, because eGFR based on creatinine measurements cannot avoid the interference of muscle mass due to differences in body mass. Therefore, obtaining measures of true glomerular filtration levels that are not confounded by muscle metabolic factors, such as inulin clearance and eGFR from radioactive elements, is particularly important in future studies (42, 43). Thus, this evidence may partially explain our results.

Meanwhile, case-control studies revealed that lower birth weight may be associated with the onset and progression of CKD in adulthood, possibly due to damage to the nephrons (44–47). The causes of impaired renal function may be related to the severity of perinatal complications, lower birth weight and shorter gestational age (48). However, due to the lack of the adolescents’ relevant medical history dating back to birth in the NHANES database, it is not known whether there is a low glomerular filtration rate secondary to birth in the underweight adolescents in the present study. Therefore, an in-depth study based on a larger sample size is urgently needed to validate the findings of this study.

There are several limitations to our study. First, the present study is a cross-sectional study based on the NHANES database, which has the inherent weakness of being unable to reveal causal relationships between exposures and outcomes. Second, although we adjusted for numerous covariates that may influence the relationship between SUA and eGFR, the lack of prior medical history of adolescents in the database may have some impact on the effect values of the final study findings. Finally, we used the latest revision of the Schwartz formula to calculate the estimated glomerular filtration rate (22), but this is not a completely accurate proxy for the true glomerular filtration rate in adolescents of all ages. Therefore, more in-depth and well-designed longitudinal study designs are needed to elucidate the relationship between the two.

## Conclusion

SUA was negatively associated with eGFR in adolescents aged 12-19 years. Furthermore, we found for the first time that SUA affects eGFR differently in adolescents with different BMIs. This effect was particularly significant in underweight adolescents. Therefore, we suggest that society and families should pay attention to the monitoring and management of BMI in adolescents, thus providing guidance for the prevention of CKD in adolescents. However, this study conclusion needs to be confirmed in further prospective studies and in a larger representative sample of underweight adolescents.

## Data availability statement

The datasets presented in this study can be found in online repositories. The names of the repository/repositories and accession number(s) can be found in the article/**Supplementary Material**.

## Ethics statement

The studies involving human participants were reviewed and approved by The ethics review board of the National Center for Health Statistics. Written informed consent to participate in this study was provided by the participants’ legal guardian/next of kin. Written informed consent was obtained from the individual(s), and minor(s)’ legal guardian/next of kin, for the publication of any potentially identifiable images or data included in this article.

## Author contributions

QT and CH had the idea for the study. QT and CH selected studies for inclusion and abstracted data. ZW, MH, YF and QT did the statistical analyses. CH and QT interpreted the data. QT wrote the first draft. MZ and QH critically revised the paper for important intellectual content. All authors contributed to the article and approved the submitted version.
